# Utilizing a Single Silica Nanospring as an Insulating Support to Characterize the Electrical Transport and Morphology of Nanocrystalline Graphite

**DOI:** 10.3390/ma12223794

**Published:** 2019-11-19

**Authors:** Peter M. Wojcik, Negar Rajabi, Haoyu Zhu, David Estrada, Paul H. Davis, Twinkle Pandhi, I. Francis Cheng, David N. McIlroy

**Affiliations:** 1 Department of Physics, University of Idaho, Moscow, ID 83844, USA; petewojcik@gmail.com (P.M.W.); pasargad23@gmail.com (N.R.); 2 Department of Chemistry, University of Idaho, Moscow, ID 83844, USA; zhu1099@vandals.uidaho.edu (H.Z.); ifcheng@uidaho.edu (I.F.C.); 3 Micron School of Materials Science and Engineering, Boise State University, Boise, ID 83725, USA; daveestrada@boisestate.edu (D.E.); pauldavis2@boisestate.edu (P.H.D.); twinklepandhi@u.boisestate.edu (T.P.); 4 Center for Advanced Energy Studies, Boise State University, Boise, ID 83706, USA; 5 Department of Physics, Oklahoma State University, Stillwater, OK 74074, USA

**Keywords:** nanocrystalline graphite, nanosprings, nanocoils, nanowires, TCOR (temperature coefficient of resistivity), Raman spectroscopy, SEM (scanning electron microscopy), TEM (transmission electron microscopy)

## Abstract

A graphitic carbon, referred to as graphite from the University of Idaho thermolyzed asphalt reaction (GUITAR), was coated in silica nanosprings and silicon substrates via the pyrolysis of commercial roofing tar at 800 °C in an inert atmosphere. Scanning electron microscopy and transmission electron microscopy images indicate that GUITAR is an agglomeration of carbon nanospheres formed by the accretion of graphitic flakes into a ~100 nm layer. Raman spectroscopic analyses, in conjunction with scanning electron microscopy and transmission electron microscopy, indicate that GUITAR has a nanocrystalline structure consisting of ~1–5 nm graphitic flakes interconnected by amorphous sp^3^ bonded carbon. The electrical resistivities of 11 single GUITAR-coated nanospring devices were measured over a temperature range of 10–80 °C. The average resistivity of all 11 devices at 20 °C was 4.3 ± 1.3 × 10^−3^ Ω m. The GUITAR coated nanospring devices exhibited an average negative temperature coefficient of resistivity at 20 °C of −0.0017 ± 0.00044 °C^−1^, which is consistent with the properties of nanocrystalline graphite.

## 1. Introduction

The carbon material dubbed graphite from the University of Idaho thermolyzed asphalt reaction (GUITAR) was first observed as the carbonaceous residue from the combustion of oil shale. Previous scanning electron microscopy (SEM) and optical images of GUITAR are consistent with a typical graphitic material [1]. X-ray photoelectron spectra indicate that the C bonding in GUITAR is a combination of sp^2^ and sp^3^ hybridized carbon [1], further supporting the conclusion that it is a form of graphite. However, GUITAR exhibits unique electrochemical properties that deviate from those of typical graphitic materials. The basal plane of GUITAR has fast, heterogeneous electron transfer kinetics and outperforms graphene, graphites, carbon nanotubes, boron-doped diamond, and diamond-like carbon [2,3]. The aqueous anodic and cathodic limits notably exceed those of other graphitic carbons, which results in better corrosion resistance relative to other graphitic materials [2,4]. These features make GUITAR an excellent candidate for use in applications such as sensors [5], energy storage and conversion [6], and water purification [3].

The unique electrochemical properties of GUITAR suggest that it is not just another form of graphite; further investigation of its morphology and electrical properties is required to classify GUITAR within the spectrum of carbon materials. The electrical resistivity and the temperature dependence on the resistivity of carbon nanocoils (NCs), graphite, and other allotropes of carbon vary greatly from allotrope to allotrope [7,8,9,10,11,12,13,14,15,16,17,18,19], thereby assisting in their identification and classification. The Raman spectra of the different carbon allotropes is equally diverse [20,21,22,23,24,25,26,27,28]. When taken in conjunction with one another, the electrical characterization and the Raman spectroscopy of GUITAR should be sufficient to identify and classify it within the spectrum of carbon allotropes.

Herein, we report on a unique approach to producing a better understanding of the morphology and electrical properties of GUITAR. GUITAR was coated onto silica nanosprings (NSs), which served as an insulating support. Then, a single GUITAR-coated NS (G-NS) device was constructed to determine the electrical properties of GUITAR. The use of silica NSs allowed us to precisely characterize the morphology of GUITAR and correlate it with its electrical properties. While this approach is not well-suited to single crystal materials, it is ideal for characterizing the electrical properties of amorphous and polycrystalline materials such as GUITAR.

## 2. Experimental

### 2.1. Silica NS Growth and GUITAR Deposition

**Figure 1:**
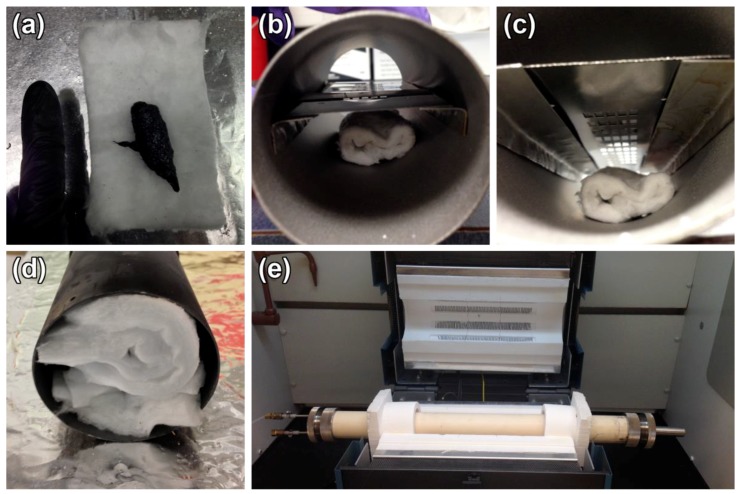
Graphite from the University of Idaho thermolyzed asphalt reaction (GUITAR) deposition experimental setup. (**a**) Five grams of asphalt on an alumina blanket; (**b**) alumina blanket with asphalt placed below a NS sample inside a cylindrical steel tube; (**c**) NS-coated surface faces down toward an alumina blanket; (**d**) endcaps of the cylindrical steel tube capped with the alumina blanket; (**e**) tube furnace with the N_2_ inlet on the left and open exhaust on the right.

Silica NSs were grown in bulk on silicon substrates using a method previously described by Wojcik et al. [29] and Wang et al. [30]. GUITAR was deposited onto the silica NS surface via the pyrolysis of commercial roofing tar (asphalt) in a tube furnace operating at 800 °C under a continuous flow of nitrogen. The deposition of GUITAR has been previously described in detail [1,4,31]. This process was modified slightly in order to deposit a thin ~100 nm coating on the NS surface. Five grams of asphalt were wrapped in an alumina blanket and placed in the center of a hollow, 10 inch long by 3 inch diameter, steel cylindrical tube (Figure 1a–c). The silicon substrate with bulk silica NSs was placed above the alumina blanket and the steel cylinder was placed in the center of a tube furnace. The furnace was ramped to 800 °C in 15 min while flowing N_2_, then cooled slowly over five hours until the furnace temperature reached ~50 °C, at which point the sample was removed.

### 2.2. Device Fabrication

**Figure 2:**
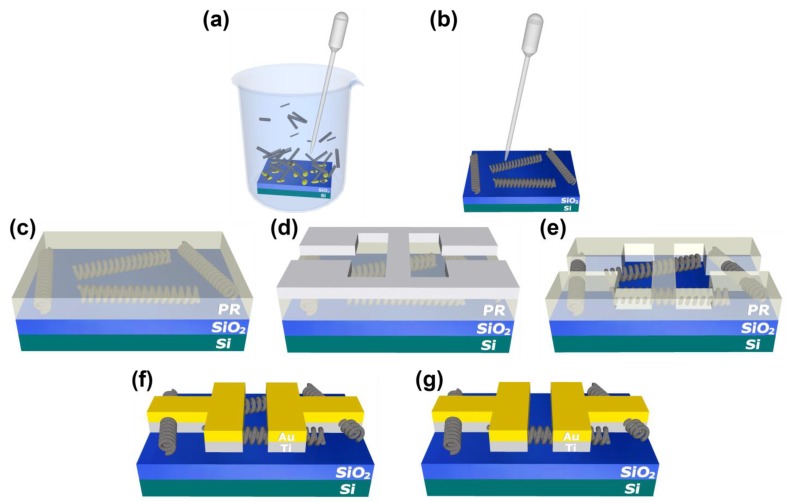
Schematic representation of the device fabrication process. (**a**) GUITAR-coated nanosprings (G-NSs) released into IPA and (**b**) drop-cast onto a SiO_2_/Si substrate. (**c**) Photoresist (PR) is spun onto the substrate. (**d**) UV light is exposed to photoresist with a chrome mask in place to create the electrode pattern. (**e**) The photoresist exposed to UV is removed with a developer. (**f**) Ti and Au are deposited via thermal evaporation and the remaining photoresist is removed in a solution of photoresist remover. (**g**) The electrode gap is cleared of G-NSs via a micromanipulator until one G-NS remains with sufficient contact.

A drop-casting method was used to deposit the G-NS on a silicon substrate with a 500 nm thick thermal oxide (SiO_2_/Si). The G-NS/isopropanol (IPA) suspension was obtained by submerging the G-NS coated silicon substrate in a beaker of IPA and vigorously agitating with a pipette, thereby releasing the G-NSs into solution (Figure 2a). Prior to drop-casting, the SiO_2_/Si substrates were cleaned by sequential soaking in acetone, IPA, and deionized water, followed by drying with N_2_. The G-NS/IPA solution was drop-cast onto the clean SiO_2_/Si and the IPA was allowed to evaporate (Figure 2b). This procedure was repeated until the desired amount of G-NSs were deposited on the SiO_2_/Si substrate. Following drop-casting, a lithographic pattern of a series of interdigitated electrodes was produced on the SiO_2_/Si substrate by a standard bilayer photolithography process using LOR3A (lift-off resist) and SPR220-4.5 photoresist. Both resists were manufactured by MicroChem Corporation. The LOR3A provided an undercut layer beneath the SPR220-4.5, which facilitated the removal of the photoresist during the lift-off process. The electrical contacts consisted of sequential layers of Ti (20 nm) and Au (200 nm) deposited by thermal evaporation. A Model 110 micromanipulator with a Model 7A probe tip (The Micromanipulator Co., Carson City, NV, USA) and an optical microscope were used to selectively remove the G-NSs along the electrode gap until one G-NS remained. Chips were fabricated with 27 interdigitated electrodes. Each electrode had a ~12,000 µm long channel to increase the probability of making Ohmic contact with a single G-NS. The yield for this design process was ~5 active devices per chip.

### 2.3. Electrical Measurements and Microscopy Equipment

Electrical measurements were acquired using a Keithley Model 2400 (Tektronix, Beaverton, OR, USA) and two Model 110 micromanipulators (The Micromanipulator Co., Carson City, NV, USA). The single G-NS devices were heated by placing the devices on a single stage thermoelectric cooler powered with an HP Model 6033A (Hewlett-Packard, Palo Alto, CA, USA) power supply. The device temperature was monitored with an Omega Model HH12 (Omega Engineering, Norwalk, CT, USA) digital thermometer. Atomic force microscopy (AFM) images were obtained with a Veeco Autoprobe Di CP-II (Veeco, Plainview, NY, USA) operating in noncontact mode with a 1 Hz scan rate. SEM images were obtained with a Zeiss Supra 35 SEM (Zeiss, Peabody, MA, USA). Transmission electron microscopy (TEM) images were obtained with a JEOL 2010J (JEOL USA, Peabody, MA, USA) operating at 200 kV.

### 2.4. Raman Spectroscopy

In order to investigate the excitation wavelength dependence of GUITAR’s Raman spectrum, Raman spectra of GUITAR thin films were collected using five different laser excitation wavelengths: 442 nm (HeCd), 488 nm and 514 nm (Ar ion), 532 nm (frequency doubled Nd:YAG), and 633 nm (HeNe). The Raman spectra at excitation wavelengths of 442 nm, 532 nm, and 633 nm were obtained with a LabRAM HR Evolution confocal Raman microscope (Horiba Scientific, Piscataway, NJ, USA) using a 100 × 0.90 NA ultrashort (210 µm) working distance Olympus objective, which produced a spot size of ~600–900 nm at the sample surface depending on the excitation wavelength. Spectra were dispersed onto a thermoelectrically cooled CCD array detector using an 1800 line/mm grating blazed for visible wavelengths. Two 30 s accumulations were averaged to maximize signal to noise and enable removal of cosmic rays from the spectra. Neutral density filters were inserted into the excitation beam until no evidence of thermal (i.e., sample heating) or nonlinear (power) effects were seen in the resultant spectra. The system used for acquisition of Raman spectra at excitation wavelengths of 488 nm and 514 nm consisted of a Coherent Innova 90-3 argon ion laser (Coherent, Santa Clara, CA, USA) and a Horiba Jobin Yvon T64000 triple monochromator (Horiba Scientific, Piscataway, NJ, USA) equipped with a 2400 line/mm grating and a liquid nitrogen cooled CCD array detector. Spectra were collected at a laser power of 200 mW with a spot size of ~100 µm, corresponding to a power density ~2 orders of magnitude lower than the Raman microscope system and, thus, well below the threshold for any thermal or power effects. To ensure the Raman spectra obtained were representative, multiple locations across multiple samples were analyzed with both Raman systems.

## 3. Results and Discussion

### 3.1. Raman Spectroscopy

**Figure 3:**
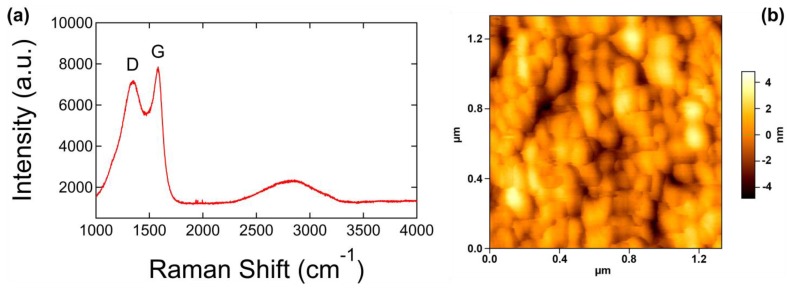
(**a**) Raman spectrum of GUITAR on a silicon substrate obtained with a 532 nm excitation source and (**b**) an AFM image of GUITAR on a silicon substrate showing GUITAR’s surface morphology, which is comprised of an agglomeration of carbon spheroids ~50 nm in diameter.

Figure 3a shows a Raman spectrum with a 532 nm excitation source of a GUITAR coating on a silicon substrate. Figure 3b shows the corresponding AFM image of the surface of GUITAR on a silicon substrate. The surface is an agglomeration of carbon spheroids ~50 nm in diameter. A discussion of the surface morphology is presented in the following section. The Raman spectrum in Figure 3a consists of two prominent peaks located at 1347 cm^−1^ and 1589 cm^−1^, as well as a broad peak centered at ~2800 cm^−1^. The peak at 1589 cm^−1^, also known as the G peak, is attributed to a phonon with E_2g_ symmetry and is associated with in-plane bond stretching of pairs of sp^2^ hybridized carbon atoms [21,32]. The wavenumber of the G peak falls in the range of 1500–1630 cm^−1^ and does not require the presence of six-fold rings, i.e., it is present for all sp^2^ bonding [32]. The peak located at 1347 cm^−1^, commonly denoted as the D peak, originates from a phonon with A_1g_ symmetry [21]. The D peak, in conjunction with the G peak, is only observed in the presence of disorder and is not present in the Raman spectra of ideal graphite [21,32]. A small, yet notable, feature is present on the shoulder of the D peak at ~1150 cm^−1^. This peak is commonly used to characterize nanocrystalline diamond and attributed to sp^3^ bonding [33,34,35,36]. However, Ferrari et al. argue that this peak must originate from an alternate chain of sp^2^ carbon atoms formed by a single hydrogen bonded to each C [37]. A previous study of GUITAR using XPS showed that the material could possibly contain alternate chains of sp^2^-bonded carbon atoms, such as C=N, which could account for the peak at ~1150 cm^−1^ [1].

Ferrari and Robertson studied the visible Raman spectra of disordered carbons and presented a three stage model of disorder from graphite to amorphous carbon that simplified the characterization of Raman spectra for all types of carbons [32]. The stages from ordered to disordered are as follows: Stage (1), graphite to nanocrystalline graphite (nc-G); Stage (2), nc-G to amorphous carbon with a small sp^3^ content (a-C); Stage (3), a-C to tetrahedral amorphous carbon with a high sp^3^ content (ta-C) [32]. Each stage has unique features in its Raman spectra, which can be used to distinguish between the three stages. Most notable in the Raman spectrum of Figure 3a are the broad peak located at ~2800 cm^−1^ and the absence of well-defined second order G or D peaks. The span of the broad peak is from ~2300–3200 cm^−1^. It is a feature that is only present in the evolution of Stage (2) of nc-G to a-C [32,38] and is a combination of the 2D, D + D′, and 2D′ bands [32,39].

**Figure 4:**
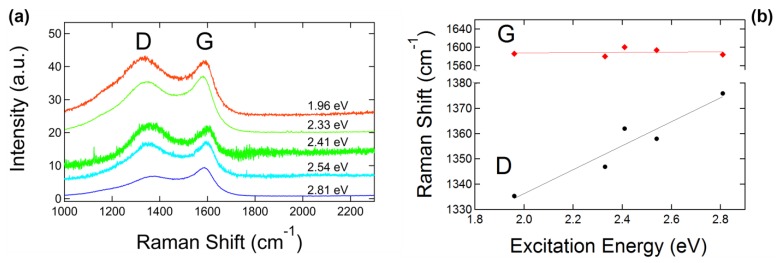
(**a**) The Raman spectra of GUITAR for a variety of excitation energies showing the prominent D and G peaks, and (**b**) the positions of the D and G peaks plotted as a function of excitation energy.

The excitation energy dependence of GUITAR’s Raman spectrum provides further evidence that GUITAR corresponds to Stage (2) carbon formation. Figure 4a shows the Raman spectra of GUITAR with 1.96 eV (633 nm), 2.33 eV (532 nm), 2.41 eV (514 nm), 2.54 eV (488 nm), and 2.81 eV (442 nm) excitation energies (wavelengths). The Raman spectrum for each excitation energy was fitted with a Lorentzian line shape for the D peak and a Breit–Wigner–Fano (BWF) line shape for the G peak. The BWF line shape function is as Equation (1),

(1) $$I\left(\omega \right)=\frac{I_{0}{\left[1+2\left(\omega -\omega_{0}\right)/Q\Gamma \right]}^{2}}{1+{\left[2\left(\omega -\omega_{0}\right)/\Gamma \right]}^{2}}$$

where I is the intensity, ω is the frequency, $I_{0}$ is the peak intensity, $\omega_{0}$ is the peak position, Γ is the full width at half maximum, and Q is the BWF coupling coefficient. A Lorentzian shape is recovered in the limit $Q^{-1}\to 0$. The asymmetry of the BWF line can account for lower frequency Raman features at ~1100 cm^−1^ and 1400 cm^−1^ without the addition of two extra peaks [32], and the combination of the BWF and Lorentzian lines is a good fit for the Raman spectral features of all types of carbons and excitation energies [32]. We also define the G peak position as $\omega_{max}$ in Equation (2):

(2) $$\omega_{max}=\omega_{0}+\frac{\Gamma }{2Q}$$

where $\omega_{0}$ lies at higher frequencies because Q is negative and is the position of the undamped mode, and is therefore higher than the apparent G peak maximum [32]. We also define the ratio of the intensity of the D and G peaks (*I*(D)/*I*(G)) using the peak heights of the D and G bands rather than the peak areas, which is common when using two Gaussian fits for the D and G peaks. The positions of the D and G peak are plotted as a function of excitation energy in Figure 4b. The vertical error bars in Figure 4b are smaller than the markers on the plot. The dispersion of the D peak as a function of excitation energy is ~48 cm^−1^/eV and is consistent with the dispersion of the Raman D peak of microcrystalline graphite [40]. The dispersion of the D peak is present in the Raman spectra of all types of carbons and has been observed to have an inverse relationship with the degree of disorder [41]. We observed that the G peak position has little to no dependence with excitation energy and remains roughly flat at ~1580 cm^−1^, as shown in Figure 4b. These results are consistent with studies of the dispersion of the Raman G band of microcrystalline graphite, where Póscik et al. observed the G peak position to be independent of excitation energies ranging from ~1 eV to 4.5 eV and at a band position of ~1580 cm^−1^ [40]. In disordered carbons, the position of the G peak is positively correlated with excitation energy, and the degree of its dispersion increases with disorder [41]. For Stage (2) carbon, the position of the G peak decreases from ~1600 cm^−1^ to 1510 cm^−1^ for nc-G to a-C [32]. Additionally, the G peak does not disperse in nc-G [41], which suggests that GUITAR lies somewhere near the beginning of Stage (2) carbon formation, characteristic of nc-G with a low sp^3^ content.

**Figure 5:**
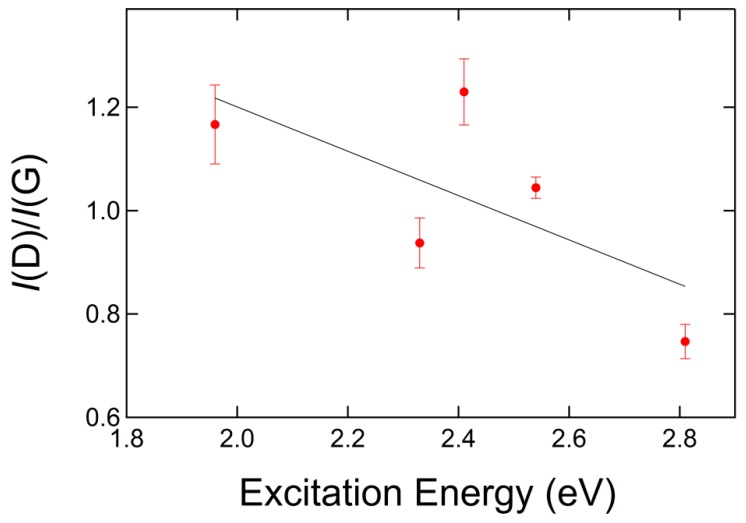
The ratio of the intensities of the D and G peaks as a function of excitation energy.

The *I*(D)/*I*(G) ratio alone cannot be used to accurately estimate the sp^3^ fraction of Stage (2) carbon. Ferrari et al. studied the Raman spectra of both an amorphization trajectory and an ordering trajectory with independent measures of the sp^3^ fractions for each stage. They found a hysteresis cycle that shows no relationship between the *I*(D)/*I*(G) ratio or the position of the G peak and the sp^3^ fraction [32]. The *I*(D)/*I*(G) ratio can, however, be used to estimate the crystalline size, *L_a_* [21]. Tuinstra and Koenig showed that the *I*(D)/*I*(G) ratio is inversely proportional to the average crystal size using the relationship, *I*(D)/*I*(G) = *C*(λ)/*L*_a_, where *C* (514 nm) ~4.4 nm [20,21,42]. In the evolution of Stage (2) carbon, the *I*(D)/*I*(G) ratio approaches zero and the Tuinstra–Koenig relation is no longer valid. Ferrari and Robertson proposed the following new relationship for a carbon nearing the end of Stage (2) and approaching that of a-C and the regime of *L*_a_ < 2 nm: *I*(D)/*I*(G) = *C*′(λ) $L_{a}^{2}$, where *C*′(514 nm) ~0.0055 [32]. Figure 5 shows the ratio of the intensities of the D and G peaks plotted as a function of excitation energy. The features of the Raman spectra to this point are characteristic of nc-G with a low sp^3^ content, which in Ferrari’s three stage disorder spectrum lies somewhere in the beginning of Stage (2), yet its exact location in the evolution of Stage (2) is undetermined. We therefore estimated the crystal size using both the Tuinstra–Koenig and the Ferrari–Robertson relationship. We found that for a 514 nm source, the GUITAR crystal size was ~1.5 nm using the Ferrari–Robertson relationship and ~3.6 nm using the Tuinstra–Koenig relationship, setting limits on the crystal size of 1.5 nm ≤ *L*_a_ ≤ 3.6 nm.

### 3.2. GUITAR Surface Morphology

**Figure 6:**
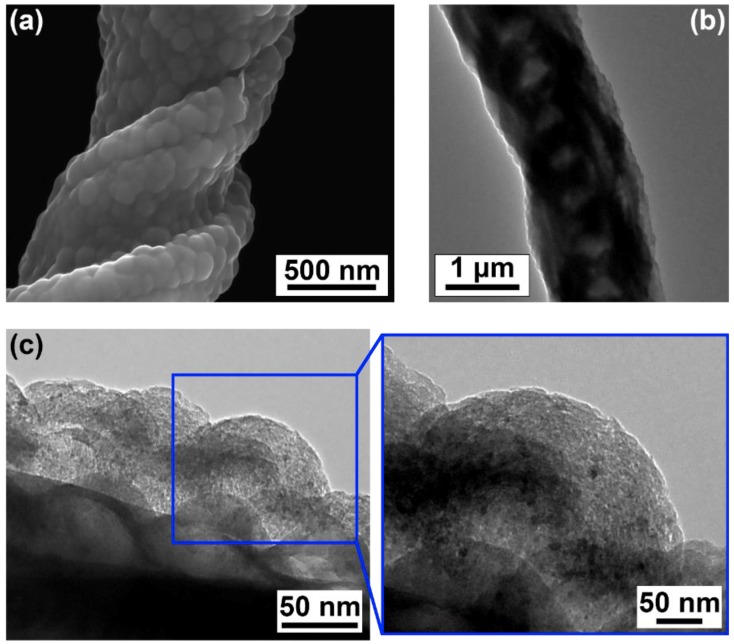
(**a**) SEM image of the surface of a G-NS. (**b**) TEM image of a single G-NS showing the GUITAR coating and the inner core of a NS. (**c**) TEM images of a single G-NS showing the interface of the NS and the GUITAR coating, and outset showing a side view of a single carbon nanosphere within the GUITAR coating.

Figure 6a is an SEM image of the G-NS surface that exhibits a pattern of smooth hemispheres ~50–100 nm in diameter. Figure 6b is a TEM image of a single G-NS. The TEM image in Figure 6c is a lateral view of the surface of a single G-NS, showing the GUITAR/NS interface with an outset showing a detailed view of one carbon nanosphere. The TEM images in Figure 6b,c show the inner core of the NS, which is comprised of several individual silica nanowires bundled together to form a larger, helical structure ~1 µm in diameter. The GUITAR coating is visible on the outer edge of the NS and the GUITAR/NS interface is well defined. Bare silicon substrates were placed alongside the bulk NS samples during the deposition of GUITAR to compare the surface morphology of GUITAR on NSs and on a flat surface. The surface morphology of GUITAR on a flat silicon substrate, as shown in the AFM image in Figure 3b, resembles that of GUITAR on a NS and confirms the presence of an agglomeration of hemispheres ~50–100 nm in diameter. TEM images of eight individual G-NSs were used to calculate the average thickness of the GUITAR coating. For each of the eight G-NSs, ~5–10 locations were chosen to measure the thickness of the coating. The total average thickness of the GUITAR coating was 87 ± 32 nm. The AFM image in Figure 3b is consistent with both the SEM and TEM images in Figure 6a–c, and these images together show that the GUITAR coating is a ~100 nm thick layer comprised of an agglomeration of irregularly shaped carbon nanospheres with diameters ranging from ~50–100 nm.

Micrometer-sized and nanometer-sized carbon-shaped spheres have been produced via a plethora of methods, and a comprehensive study of the variety of morphologies has been conducted. Inagaki et al. proposed that spherical carbon bodies could be classified into three categories based on their nanometric texture, e.g., the concentric, radial, and random arrangement of the carbon layers [43]. Serp et al. further classified spherical carbons with three additional categories based on their size: carbon onions and the C_n_ family, with diameters ranging from 2–20 nm; carbon nanospheres that exhibit a small degree of graphitization and that have diameters ranging from 50 nm to 1 µm; and carbon beads that range in diameter from one to several microns [44]. Inagaki classified carbon nanospheres produced from the thermal decomposition of hydrocarbon gases as carbon blacks and proposed a concentric arrangement of carbon layers [43]. This concentric texture has been observed with TEM and shows that carbon nanospheres are composed of graphitic layers with unclosed graphitic flakes on their surfaces [45,46]. Carbon nanospheres are also generally observed as an agglomeration of carbon spheres with varying diameters [45,47,48,49,50]. The coalescence and accretion of carbon nanospheres was observed by Nieto-Marquez et al. via a catalytic growth method [49], Kang and Wang via catalytic carbonization after treatment in acetone [45], and by Jin et al. via the pyrolysis of a variety of hydrocarbons in the absence of a catalyst [48]. The accretion of carbon nanospheres can be attributed to the high surface reactivity resulting from dangling bonds on the unclosed graphitic flakes residing on the surface of the spheres [45,46,49].

The outset in Figure 6c shows a detailed view of an individual carbon nanosphere on a NS. The morphology of its outer surface is nonuniform and shows that the sphere’s surface is comprised of unclosed graphitic flakes. Upon further inspection of the surface of the individual carbon nanosphere, we found that the flakes are nonuniform disks, which vary in diameter from ~1–5 nm. The sizes of these flakes are consistent with estimations of crystal size according to an analysis of GUITAR’s Raman spectra, which showed that 1.5 nm ≤ *L*_a_ ≤ 3.6 nm, and the *I*(D)/*I*(G) ratio (Figure 5), which also shows that GUITAR is characteristic of a material with a low degree of graphitization. All of these features demonstrate that the building block of the GUITAR coating is a carbon nanosphere, per the description by Serp et al. and Inagaki et al., which agglomerate to form a film or coating in the case of the NSs.

### 3.3. Electrical Characteristics

**Figure 7:**
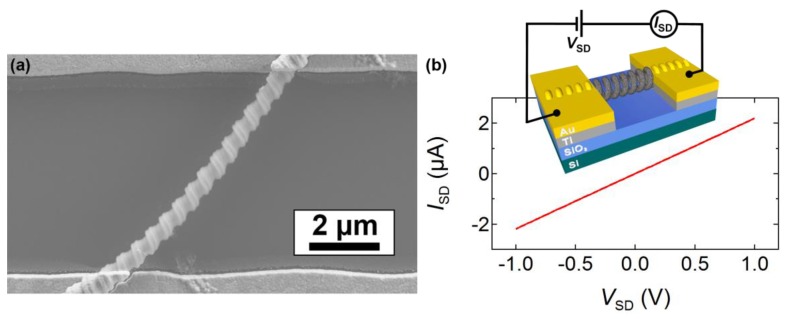
(**a**) SEM image of a single G-NS between two source–drain Ti/Au electrodes, and (**b**) the Ohmic *I*_SD_*–V*_SD_ curve of a typical G-NS device and an inset showing a three-dimensional representation of the device and the corresponding electrical diagram.

Figure 7a shows an SEM image of a typical single G-NS device spanning a ~6 µm gap between two Ti/Au source-drain electrodes. Figure 7b shows a typical two-point probe source–drain current–voltage (*I*_SD_–*V*_SD_) curve for a single G-NS device at room temperature and atmospheric pressure. The inset in Figure 7b shows a three-dimensional schematic and electrical diagram of the single G-NS device. The *I*_SD_–*V*_SD_ curve is linear and indicative of Ohmic contacts. All of the devices tested in this work displayed Ohmic behavior. The total resistance ($R_{T}$) obtained from the two-point *I*_SD_–*V*_SD_ measurement is the sum of the resistance of the single G-NS ($R_{G-\mathrm{NS}}$) and of the two contact resistances ($R_{C})$ at the GUITAR–Ti/Au interface on each end: $R_{T}=R_{G-\mathrm{NS}}+2R_{C}$. Previous studies on the contact resistance of single- and multilayered graphene to Ti/Au metal contacts have reported contact resistances of <250 Ω for multilayered graphene (~50 layers) [51], and ~165 Ω for single-layered graphene [52]. A comparison of the contact resistances for Ni contacts on graphene and highly ordered pyrolytic graphite (HOPG) has shown that the measurements are approximately two orders of magnitude higher for Ni contacts on graphene than on HOPG. This greater contact resistance is attributed to the higher charge density in HOPG [51]. Additionally, Venugopal et al. have shown that the contact resistance for metal contacts on multilayered graphene decreases as the number of graphene layers increases [51]; this is because increasing the number of graphene layers results in increased carrier concentration, until the structure approaches that of HOPG, i.e., becomes more metallic. Since the GUITAR coating is characteristic of a disordered multilayered graphitic material, it must have a high charge density, representative of multilayered graphene and HOPG. Therefore, the contact resistance of the GUITAR–Ti/Au is small (<250 Ω), or ~3 orders of magnitude smaller than the total resistance of a single G-NS (~500 kΩ), and therefore can be neglected.

The single G-NS devices failed when the magnitude of *I*_SD_ exceeded ~10 µA. Care was taken to avoid applying a large current by keeping the magnitude of *V*_SD_ below 3 V. A gate voltage was applied to the G-NS via a back-gate. *I*_SD_*–V*_SD_ curves were measured while the back-gate voltage was held constant. There was no observable change in the resistance for back-gate voltages ranging from −20 V to 20 V, suggesting that the device did not exhibit a field effect, and therefore confirming that it is not semiconducting.

Eleven single G-NS devices were used to determine the average resistivity and conductivity of the GUITAR coating. The resistivity (ρ) of each device was calculated using Equation (3):

(3) $$\rho =R\frac{A}{L}$$

where R is the average resistance of the device, A is the cross-sectional area of the GUITAR coating on the NS, and L is the length of the G-NS. For each single G-NS device, the slopes of five *I*_SD_*–V*_SD_ curves from ±1V were taken at 20 °C and used to calculate the average resistance for each device. The cross-sectional area of the GUITAR coating was determined from the average thickness of the GUITAR coating calculated from the TEM images, as described in Section 3.2. SEM images of each device were used to determine the radius and length of the NS. The helical pitch of an individual NS is random and uncontrollable during the growth process. Therefore, the helical pitches of the single G-NS devices are also random. However, the helical pitch of the single G-NS devices in this study were well-defined and constant along the entire span of the electrode gap, meaning the G-NS can be represented as a helix with constant radius in the active region of each device, which allowed for the calculation of the wire diameter and length of each G-NS. In some instances, the pitch of the G-NS was small, so that adjacent coils are in contact with one another, creating a closed coil along the entire length of its active area in the device. In this case, the G-NS was treated as a wire and an average diameter was calculated over the pitch of two adjacent coils. The average resistivity calculated from the slopes of five *I*_SD_*–V*_SD_ curves at 20 °C for each device are shown in Table 1. The total average resistivity of the 11 single G-NS devices at 20 °C is 4.3 ± 1.3 × 10^−3^ Ω m.

**Figure 8:**
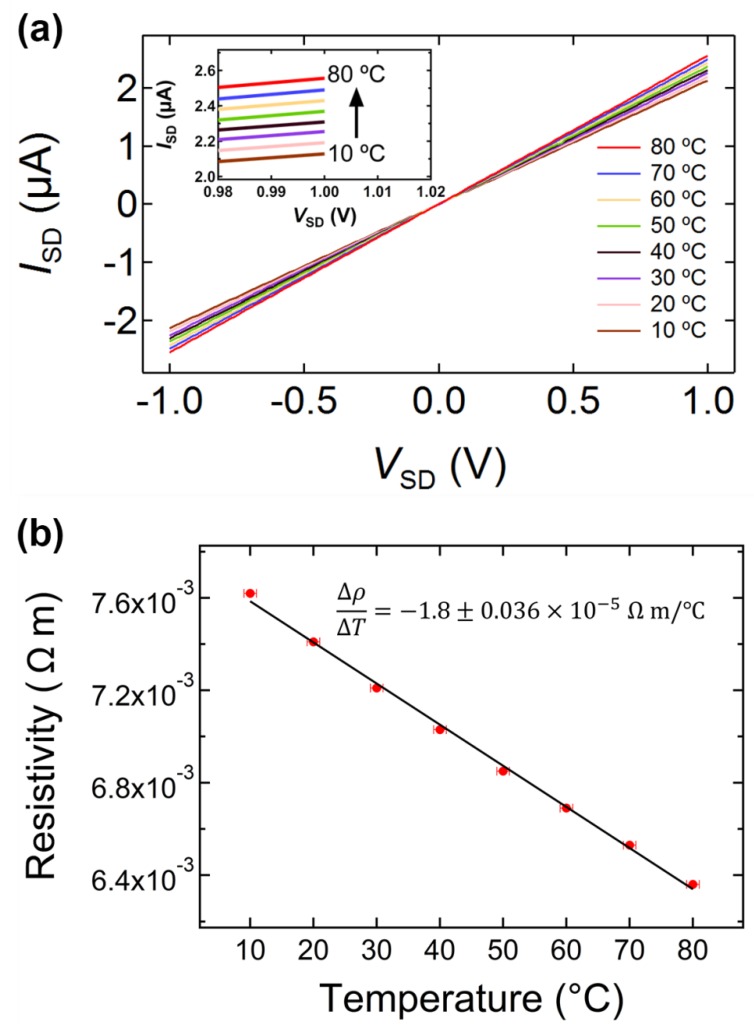
(**a**) Temperature dependent *I*_SD_–*V*_SD_ curves of a single G-NS (device 3) from 10–80 °C, where the inset is an expanded view of the upper range of *I*_SD_–*V*_SD_, and (**b**) the resistivity vs. temperature profile for the same single G-NS, which displays a negative linear slope.

The temperature-dependent *I*_SD_*–V*_SD_ curves of a single G-NS (device 3) from 10–80 °C in increments of 10 °C are displayed in Figure 8a. The inset in Figure 8a shows the upper range of the *I*_SD_*–V*_SD_ plot and the device’s linear change in resistance as a function of temperature. Note that the *I*_SD_*–V*_SD_ curve remains Ohmic across the entire temperature range. The slopes of five *I*_SD_*–V*_SD_ curves were used to calculate the average resistivity at each temperature, which are presented in Figure 8b. The vertical errors bars are smaller than the markers used in the plot. The resistivity of the device is linear with respect to temperature and decreases at ~ −1.8 × 10^−5^ Ω m/°C, i.e., has a negative slope.

The linear relationship between resistivity and temperature was used to calculate the negative temperature coefficient of resistivity (TCOR) at 20 °C. The resistivity of the G-NS can be expressed as a function of the temperature (T) as Equation (4):

(4) $$\rho \left(T\right)=\rho_{0}\;\left[1+\alpha_{0}\left(T-T_{0}\right)\right]$$

where $T_{0}$ is the reference temperature at 20 °C, $\rho_{0}$ is the resistivity at the reference temperature, and $\alpha_{0}$ is the TCOR at 20 °C. For each of the 11 single G-NS devices, the slope of the resistivity vs. temperature profiles (Δρ/ΔT) were obtained from a linear fit of the data, as shown in Figure 8b. The resistivity at the reference temperature of 20 °C for each device (Table 1) and Δρ/ΔT were used to calculate $\alpha_{0}$ with Equation (5):

(5) $$\alpha_{0}=\left(\Delta \rho /\Delta T\right)\rho_{0}{}^{-1}.$$

All of the single G-NS devices displayed equivalent temperature dependence, as shown in Figure 8a,b and Table 2. The slope of the resistivity vs. temperature profile, the resistivity at the reference temperature ($\rho_{0}$), and the calculated TCOR for each device are shown in Table 2. The average TCOR of the 11 single G-NS devices at 20 °C was −0.0017 ± 0.00044 °C^−1^.

The electrical resistivity and, in particular, the temperature dependency of the resistivity of carbon NCs, graphite, and other allotropes of carbon are well-known and have been studied extensively [7,8,9,10,11,12,13,14,15,16,17,18,19]. At and near room temperature, the temperature dependence of the resistivity for conductive carbons is linear and the TCOR can be calculated using Equation (5). The calculated TCOR of a variety of conductive carbons are summarized in Table 3. Many authors have reported the temperature dependent resistivities for large temperature ranges (~0 K–2000 K), but they did not specifically calculate the TCOR in the room temperature regime. All of the TCOR values listed in Table 3 except for GUITAR were extrapolated from the linear regime of the resistivity vs. temperature plots near room temperature and for ΔT ~100 °C.

The negative temperature dependent resistivity of G-NS is consistent with GUITAR being a graphitic form of carbon. The measured resistivity of GUITAR corresponds with measurements of the resistivity of similarly highly disordered carbon allotropes at room temperature, and it is on the order of the resistivities of an a-C thin film (70 nm) [7], nanodiamond-derived carbon onion electrodes [53], and carbon black [9]. The resistivity of GUITAR is also within an order of magnitude of the resistivities of graphitized soot [9] and carbon NCs [18] (Table 3). The TEM images of GUITAR in Figure 6 are similar to TEM images of carbon black [54] and graphitized soot [55,56], which is indicative of similar morphologies and chemical compositions, i.e., a low degree of graphitization. The higher conductivities of the remainder of the carbon materials in Table 3 scale with their degree of graphitization.

Using the TCOR alone from Table 3, it is difficult to distinguish a pure graphite from more disordered allotropes of carbon, such as a-C. It is therefore important to consider both the resistivity and the TCOR of GUITAR when classifying it within the spectrum of graphitic materials. GUITAR’s negative TCOR near room temperature corresponds with all of the carbon allotropes listed in Table 3 and confirms its classification as a graphitic semimetal material. The Raman spectra, SEM, and TEM images; the negative TCOR at and near room temperature; and the resistivity of GUITAR confirm that it is a graphitic semimetal composed of nc-G.

## 4. Conclusions

We used a combination of Raman spectroscopy, SEM, and TEM images, and the electrical characterization of 11 single G-NS devices to study the nanostructure, surface morphology, electrical resistivity, and negative TCOR of GUITAR in order to classify GUITAR within the spectrum of carbon materials. The Raman spectra of GUITAR were consistent with nc-G, in that it had low sp^3^ content with an estimated crystalline size of ~1.5–3.6 nm. SEM and TEM images show that GUITAR is an agglomeration of carbon nanospheres, where their surface is comprised of unclosed graphitic flakes ~1–5 nm in size. The electrical properties of GUITAR, as determined from the electrical measurements of 11 single G-NS devices, demonstrate that it is a semimetal and that it has properties consistent with those of nc-G. With this study, we have definitively identified GUITAR as a nanocrystalline form of graphite where the sp^2^ bonded graphite nanocrystals are connected via sp^3^ bonding. Finally, we have demonstrated the utility of silica NSs as an insulating support for measuring the electrical properties of amorphous and polycrystalline materials, or in this case, GUITAR.

## Figures and Tables

**Table 1:** The average resistivity of the 11 single G-NS devices calculated from the slopes of five *I*_SD_*–V*_SD_ curves at 20 °C for each device.

Device	Average Resistivity (Ω m) at 20 °C
1	3.1 ± 0.62 × 10^−3^
2	20 ± 3.8 × 10^−3^
3	7.4 ± 1.4 × 10^−3^
4	3.6 ± 0.75 × 10^−3^
5	2.1 ± 0.62 × 10^−3^
6	4.9 ± 1.1 × 10^−3^
7	1.6 ± 0.18 × 10^−3^
8	1.3 ± 0.25 × 10^−3^
9	1.2 ± 0.19 × 10^−3^
10	1.6 ± 0.34 × 10^−3^
11	0.92 ± 0.16 × 10^−3^

**Table 2:** The slope of the resistivity vs. temperature profile (Δρ/ΔT), the resistivity at the reference temperature ($\rho_{0}$), and the calculated TCOR for each device.

Device	Δρ/ΔT (Ω m/°C)	$\rho_{0}$ (Ω m)	TCOR (°C^−1^)
1	−8.9 ± 1.2 × 10^−6^	3.1 ± 0.62 × 10^−3^	−2.9 ± 0.69 × 10^−3^
2	−5.0 ± 1.5 × 10^−5^	20 ± 3.8 × 10^−3^	−2.5 ± 0.88 × 10^−3^
3	−1.8 ± 0.036 × 10^−5^	7.4 ± 1.4 × 10^−3^	−2.4 ± 0.46 × 10^−3^
4	−3.1 ± 0.32 × 10^−6^	3.6 ± 0.75 × 10^−3^	−0.87 ± 0.20 × 10^−3^
5	−3.7 ± 0.097 × 10^−6^	2.1 ± 0.62 × 10^−3^	−1.8 ± 0.52 × 10^−3^
6	−7.9 ± 0.36 × 10^−6^	4.9 ± 1.1 × 10^−3^	−1.6 ± 0.37 × 10^−3^
7	−2.3 ± 0.032 × 10^−6^	1.6 ± 0.18 × 10^−3^	−1.4 ± 0.17 × 10^−3^
8	−1.6 ± 0.043 × 10^−6^	1.3 ± 0.25 × 10^−3^	−1.2 ± 0.24 × 10^−3^
9	−1.3 ± 0.056 × 10^−6^	1.2 ± 0.19 × 10^−3^	−1.0 ± 0.17 × 10^−3^
10	−2.2 ± 0.049 × 10^−6^	1.6 ± 0.34 × 10^−3^	−1.4 ± 0.29 × 10^−3^
11	−1.2 ± 0.069 × 10^−6^	0.92 ± 0.16 × 10^−3^	−1.3 ± 0.24 × 10^−3^

**Table 3:** Comparison of the resistivities, conductivities, and TCOR values for a variety of materials, including GUITAR.

Material	Resistivity ρ (Ω m) at 20 °C	Conductivity σ (S/m) at 20 °C	Temperature Coefficient at 20 °C (°C^−1^)	Reference
GUITAR	4.3 × 10^−3^	2.3 × 10^2^	−0.0017	This Study
Carbon Onions	2.5 × 10^−3^	4.0 × 10^2^	N/A	[53]
Carbon Black	1.7 × 10^−3^	6.0 × 10^2^	−0.00094*	[9]
a-C (70 nm thick)	1.0 × 10^−3^	1.0 × 10^3^	N/A	[7]
Graphitized Soot	3.3 × 10^−4^	3.0 × 10^3^	−0.0014*	[9]
Carbon NC	1.9 × 10^−4^	5.3 × 10^3^	−0.0012*	[18]
POCO Graphite AF	9.6 × 10^−5^	1.0 × 10^4^	−0.0023*	[9]
Lampblack Graphite	5.5 × 10^−5^	1.8 × 10^4^	−0.0013*	[10]
Carbon	4.5 × 10^−5^	2.2 × 10^4^	−0.00040*	[12]
Grade AGOT Graphite	1.0 × 10^−5^	9.7 × 10^4^	−0.0016*	[10]
Natural Graphite	9.8 × 10^−6^	1.0 × 10^5^	−0.0010*	[10]
Grade CS Graphite	7.7 × 10^−6^	1.3 × 10^5^	−0.0017*	[10]
Acheson Graphite	6.3 × 10^−6^	1.6 × 10^5^	−0.0011*	[12]
Carbon NC (as grown)	3.6 × 10^−6^	2.8 × 10^5^	−0.0015*	[19]
Carbon NC (annealed)	4.1 × 10^−7^	2.4 × 10^6^	−0.00072*	[19]
